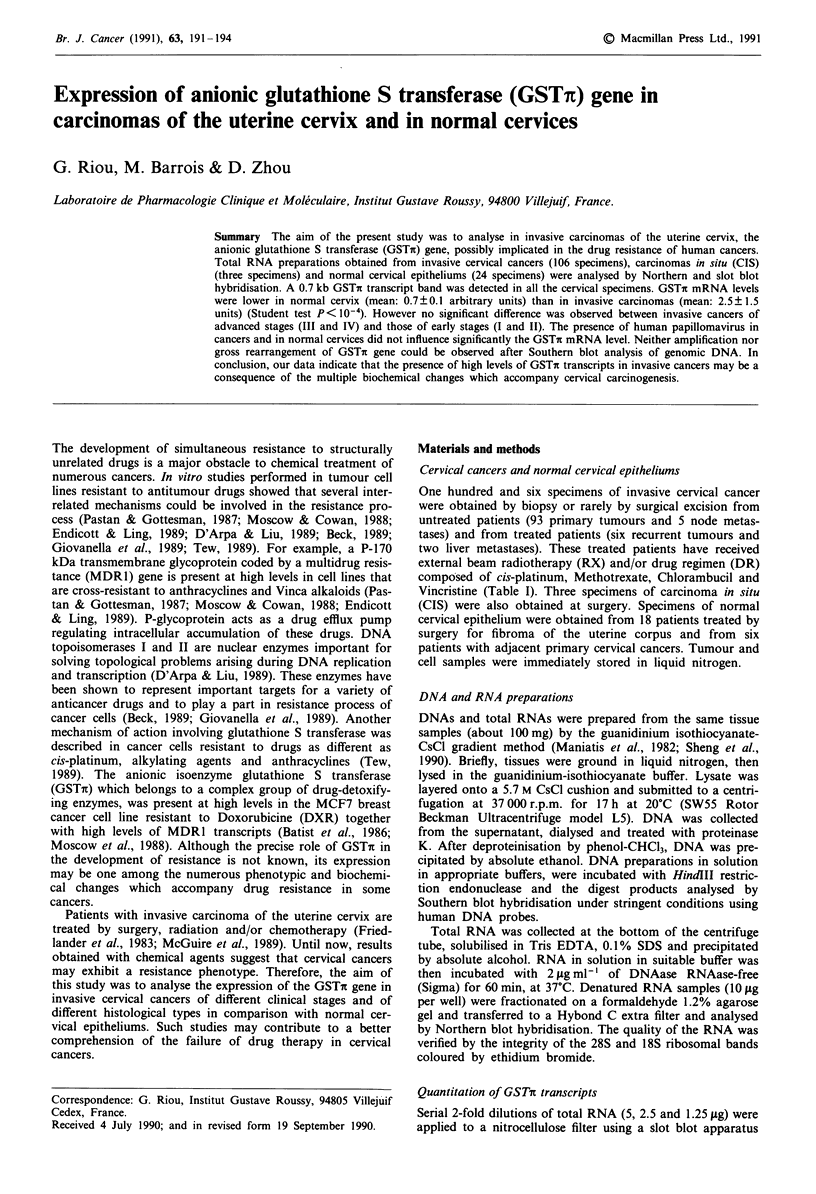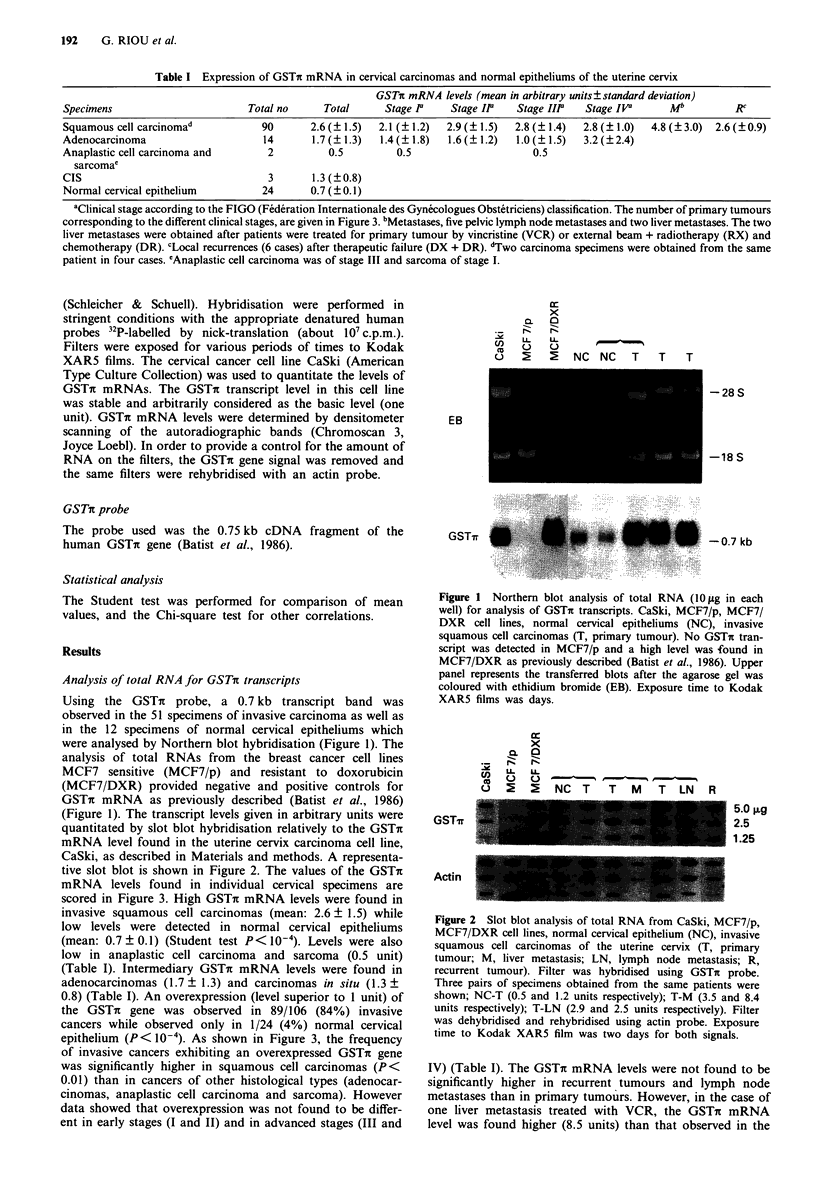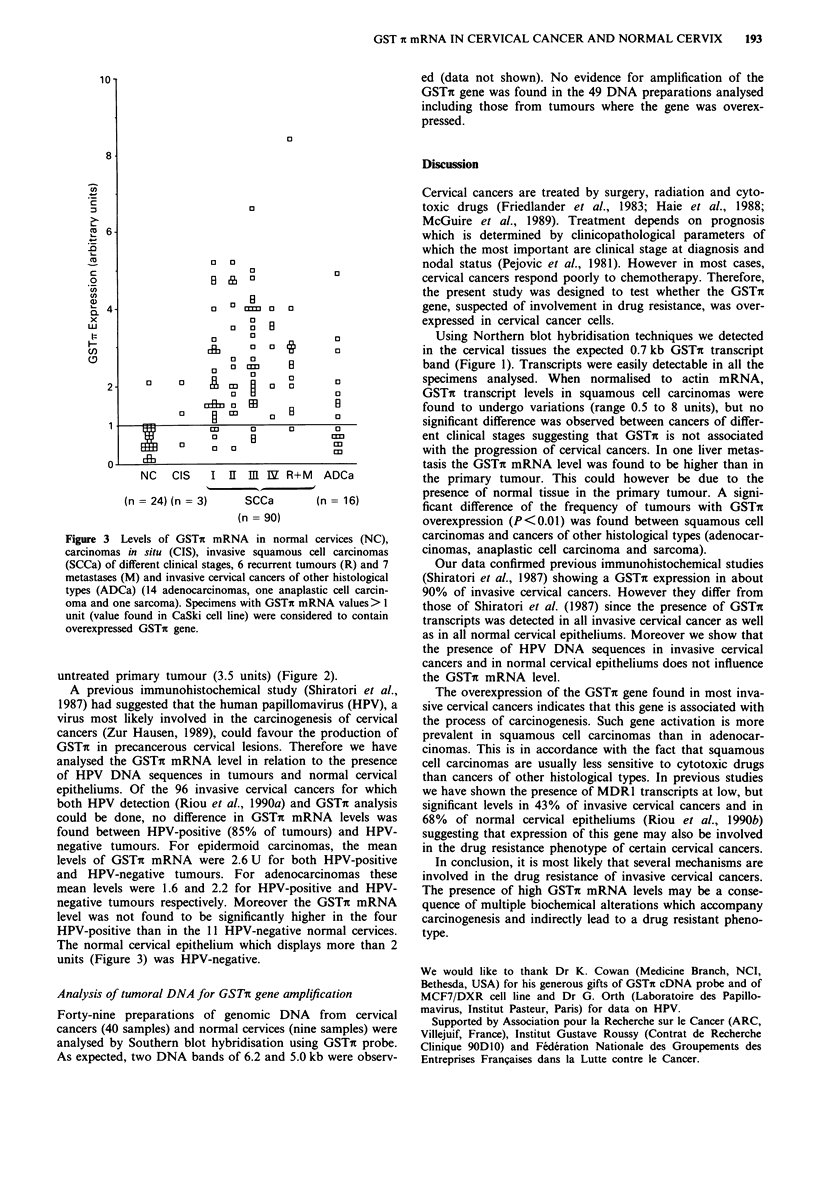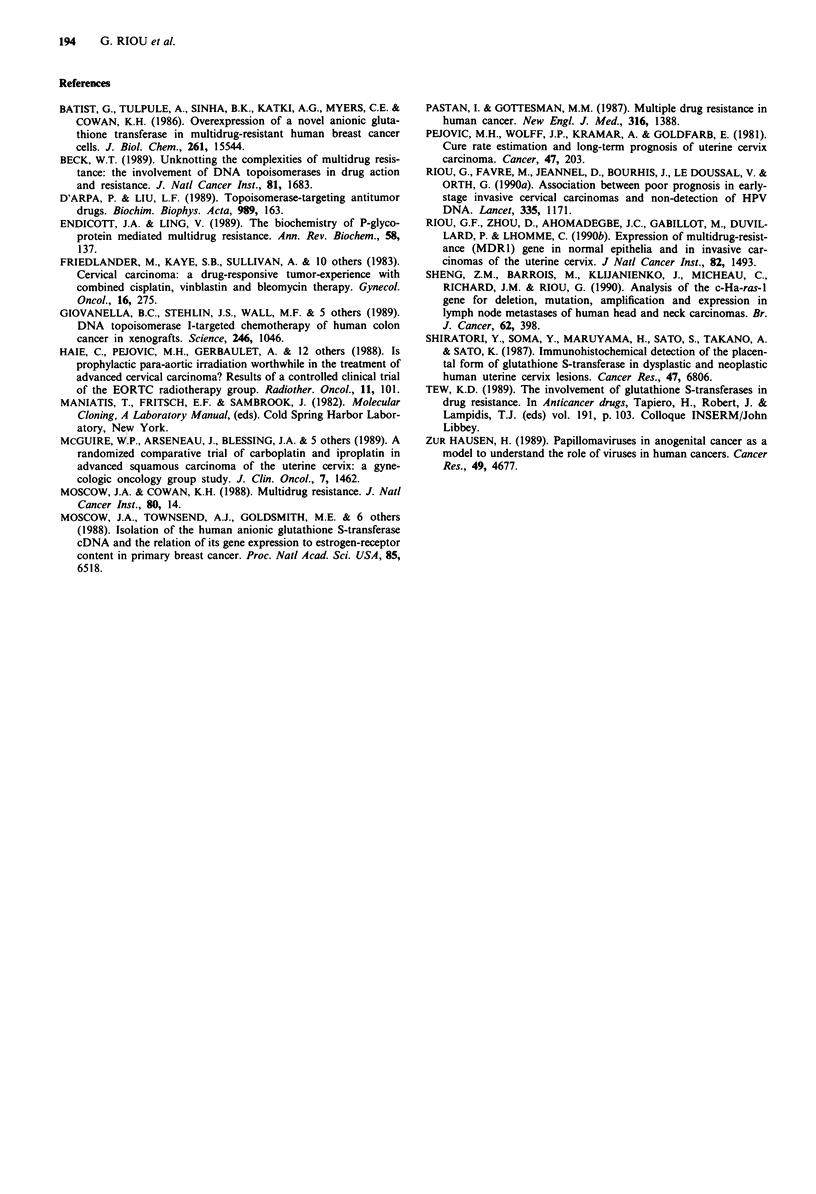# Expression of anionic glutathione S transferase (GST pi) gene in carcinomas of the uterine cervix and in normal cervices.

**DOI:** 10.1038/bjc.1991.47

**Published:** 1991-02

**Authors:** G. Riou, M. Barrois, D. Zhou

**Affiliations:** Laboratoire de Pharmacologie Clinique et Moléculaire, Institut Gustave Roussy, Villejuif, France.

## Abstract

**Images:**


Br. J. Cancer (1991), 63, 191–194 © Macmillan Press Ltd., 1991

### Expression of anionic glutathione S transferase (GSTπ) gene in carcinomas of the uterine cervix and in normal cervices

G. Riou, M. Barrois & D. Zhou

*Laboratoire de Pharmacologie Clinique et Moléculaire, Institut Gustave Roussy, 94800 Villejuif, France.*

**Summary** The aim of the present study was to analyse in invasive carcinomas of the uterine cervix, the anionic glutathione S transferase (GSTπ) gene, possibly implicated in the drug resistance of human cancers. Total RNA preparations obtained from invasive cervical cancers (106 specimens), carcinomas *in situ* (CIS) (three specimens) and normal cervical epitheliums (24 specimens) were analysed by Northern and slot blot hybridisation. A 0.7 kb GSTπ transcript band was detected in all the cervical specimens. GSTπ mRNA levels were lower in normal cervix (mean: 0.7±0.1 arbitrary units) than in invasive carcinomas (mean: 2.5±1.5 units) (Student test $P<10^{-4}$). However no significant difference was observed between invasive cancers of advanced stages (III and IV) and those of early stages (I and II). The presence of human papillomavirus in cancers and in normal cervices did not influence significantly the GSTπ mRNA level. Neither amplification nor gross rearrangement of GSTπ gene could be observed after Southern blot analysis of genomic DNA. In conclusion, our data indicate that the presence of high levels of GSTπ transcripts in invasive cancers may be a consequence of the multiple biochemical changes which accompany cervical carcinogenesis.

The development of simultaneous resistance to structurally unrelated drugs is a major obstacle to chemical treatment of numerous cancers. *In vitro* studies performed in tumour cell lines resistant to antitumour drugs showed that several inter-related mechanisms could be involved in the resistance process (Pastan & Gottesman, 1987; Moscow & Cowan, 1988; Endicott & Ling, 1989; D'Arpa & Liu, 1989; Beck, 1989; Giovanella *et al.*, 1989; Tew, 1989). For example, a P-170 kDa transmembrane glycoprotein coded by a multidrug resistance (MDR1) gene is present at high levels in cell lines that are cross-resistant to anthracyclines and Vinca alkaloids (Pastan & Gottesman, 1987; Moscow & Cowan, 1988; Endicott & Ling, 1989). P-glycoprotein acts as a drug efflux pump regulating intracellular accumulation of these drugs. DNA topoisomerases I and II are nuclear enzymes important for solving topological problems arising during DNA replication and transcription (D'Arpa & Liu, 1989). These enzymes have been shown to represent important targets for a variety of anticancer drugs and to play a part in resistance process of cancer cells (Beck, 1989; Giovanella *et al.*, 1989). Another mechanism of action involving glutathione S transferase was described in cancer cells resistant to drugs as different as *cis*-platinum, alkylating agents and anthracyclines (Tew, 1989). The anionic isoenzyme glutathione S transferase (GSTπ) which belongs to a complex group of drug-detoxifying enzymes, was present at high levels in the MCF7 breast cancer cell line resistant to Doxorubicine (DXR) together with high levels of MDR1 transcripts (Batist *et al.*, 1986; Moscow *et al.*, 1988). Although the precise role of GSTπ in the development of resistance is not known, its expression may be one among the numerous phenotypic and biochemical changes which accompany drug resistance in some cancers.

Patients with invasive carcinoma of the uterine cervix are treated by surgery, radiation and/or chemotherapy (Friedlander *et al.*, 1983; McGuire *et al.*, 1989). Until now, results obtained with chemical agents suggest that cervical cancers may exhibit a resistance phenotype. Therefore, the aim of this study was to analyse the expression of the GSTπ gene in invasive cervical cancers of different clinical stages and of different histological types in comparison with normal cervical epitheliums. Such studies may contribute to a better comprehension of the failure of drug therapy in cervical cancers.

Correspondence: G. Riou, Institut Gustave Roussy, 94805 Villejuif Cedex, France.
Received 4 July 1990; and in revised form 19 September 1990.

### Materials and methods

### *Cervical cancers and normal cervical epitheliums*

One hundred and six specimens of invasive cervical cancer were obtained by biopsy or rarely by surgical excision from untreated patients (93 primary tumours and 5 node metastases) and from treated patients (six recurrent tumours and two liver metastases). These treated patients have received external beam radiotherapy (RX) and/or drug regimen (DR) composed of *cis*-platinum, Methotrexate, Chlorambucil and Vincristine (Table I). Three specimens of carcinoma *in situ* (CIS) were also obtained at surgery. Specimens of normal cervical epithelium were obtained from 18 patients treated by surgery for fibroma of the uterine corpus and from six patients with adjacent primary cervical cancers. Tumour and cell samples were immediately stored in liquid nitrogen.

### *DNA and RNA preparations*

DNAs and total RNAs were prepared from the same tissue samples (about 100 mg) by the guanidinium isothiocyanate-CsCl gradient method (Maniatis *et al.*, 1982; Sheng *et al.*, 1990). Briefly, tissues were ground in liquid nitrogen, then lysed in the guanidinium-isothiocyanate buffer. Lysate was layered onto a 5.7 M CsCl cushion and submitted to a centrifugation at 37 000 r.p.m. for 17 h at 20°C (SW55 Rotor Beckman Ultracentrifuge model L5). DNA was collected from the supernatant, dialysed and treated with proteinase K. After deproteinisation by phenol-$CHCl_3$, DNA was precipitated by absolute ethanol. DNA preparations in solution in appropriate buffers, were incubated with *Hind*III restriction endonuclease and the digest products analysed by Southern blot hybridisation under stringent conditions using human DNA probes.

Total RNA was collected at the bottom of the centrifuge tube, solubilised in Tris EDTA, 0.1% SDS and precipitated by absolute alcohol. RNA in solution in suitable buffer was then incubated with 2 $\mu g\,ml^{-1}$ of DNAase RNAase-free (Sigma) for 60 min, at 37°C. Denatured RNA samples (10 µg per well) were fractionated on a formaldehyde 1.2% agarose gel and transferred to a Hybond C extra filter and analysed by Northern blot hybridisation. The quality of the RNA was verified by the integrity of the 28S and 18S ribosomal bands coloured by ethidium bromide.

### *Quantitation of GSTπ transcripts*

Serial 2-fold dilutions of total RNA (5, 2.5 and 1.25 µg) were applied to a nitrocellulose filter using a slot blot apparatus

**Table I** Expression of GSTπ mRNA in cervical carcinomas and normal epitheliums of the uterine cervix

| | | *GSTπ mRNA levels (mean in arbitrary units ± standard deviation)* | | | | | | |
|---|---|---|---|---|---|---|---|---|
| *Specimens* | *Total no* | *Total* | *Stage I*[a] | *Stage II*[a] | *Stage III*[a] | *Stage IV*[a] | *M*[b] | *R*[c] |
| Squamous cell carcinoma[d] | 90 | 2.6 (±1.5) | 2.1 (±1.2) | 2.9 (±1.5) | 2.8 (±1.4) | 2.8 (±1.0) | 4.8 (±3.0) | 2.6 (±0.9) |
| Adenocarcinoma | 14 | 1.7 (±1.3) | 1.4 (±1.8) | 1.6 (±1.2) | 1.0 (±1.5) | 3.2 (±2.4) | | |
| Anaplastic cell carcinoma and sarcoma[e] | 2 | 0.5 | 0.5 | | 0.5 | | | |
| CIS | 3 | 1.3 (±0.8) | | | | | | |
| Normal cervical epithelium | 24 | 0.7 (±0.1) | | | | | | |

[a]Clinical stage according to the FIGO (Fédération Internationale des Gynécologues Obstétriciens) classification. The number of primary tumours corresponding to the different clinical stages, are given in Figure 3. [b]Metastases, five pelvic lymph node metastases and two liver metastases. The two liver metastases were obtained after patients were treated for primary tumour by vincristine (VCR) or external beam + radiotherapy (RX) and chemotherapy (DR). [c]Local recurrences (6 cases) after therapeutic failure (DX + DR). [d]Two carcinoma specimens were obtained from the same patient in four cases. [e]Anaplastic cell carcinoma was of stage III and sarcoma of stage I.

(Schleicher & Schuell). Hybridisation were performed in stringent conditions with the appropriate denatured human probes $^{32}$P-labelled by nick-translation (about $10^7$ c.p.m.). Filters were exposed for various periods of times to Kodak XAR5 films. The cervical cancer cell line CaSki (American Type Culture Collection) was used to quantitate the levels of GSTπ mRNAs. The GSTπ transcript level in this cell line was stable and arbitrarily considered as the basic level (one unit). GSTπ mRNA levels were determined by densitometer scanning of the autoradiographic bands (Chromoscan 3, Joyce Loebl). In order to provide a control for the amount of RNA on the filters, the GSTπ gene signal was removed and the same filters were rehybridised with an actin probe.

### *GSTπ probe*

The probe used was the 0.75 kb cDNA fragment of the human GSTπ gene (Batist *et al.*, 1986).

### *Statistical analysis*

The Student test was performed for comparison of mean values, and the Chi-square test for other correlations.

### Results

### *Analysis of total RNA for GSTπ transcripts*

Using the GSTπ probe, a 0.7 kb transcript band was observed in the 51 specimens of invasive carcinoma as well as in the 12 specimens of normal cervical epitheliums which were analysed by Northern blot hybridisation (Figure 1). The analysis of total RNAs from the breast cancer cell lines MCF7 sensitive (MCF7/p) and resistant to doxorubicin (MCF7/DXR) provided negative and positive controls for GSTπ mRNA as previously described (Batist *et al.*, 1986) (Figure 1). The transcript levels given in arbitrary units were quantitated by slot blot hybridisation relatively to the GSTπ mRNA level found in the uterine cervix carcinoma cell line, CaSki, as described in Materials and methods. A representative slot blot is shown in Figure 2. The values of the GSTπ mRNA levels found in individual cervical specimens are scored in Figure 3. High GSTπ mRNA levels were found in invasive squamous cell carcinomas (mean: 2.6 ± 1.5) while low levels were detected in normal cervical epitheliums (mean: 0.7 ± 0.1) (Student test $P < 10^{-4}$). Levels were also low in anaplastic cell carcinoma and sarcoma (0.5 unit) (Table I). Intermediary GSTπ mRNA levels were found in adenocarcinomas (1.7 ± 1.3) and carcinomas *in situ* (1.3 ± 0.8) (Table I). An overexpression (level superior to 1 unit) of the GSTπ gene was observed in 89/106 (84%) invasive cancers while observed only in 1/24 (4%) normal cervical epithelium ($P < 10^{-4}$). As shown in Figure 3, the frequency of invasive cancers exhibiting an overexpressed GSTπ gene was significantly higher in squamous cell carcinomas ($P <$ 0.01) than in cancers of other histological types (adenocarcinomas, anaplastic cell carcinoma and sarcoma). However data showed that overexpression was not found to be different in early stages (I and II) and in advanced stages (III and

**Figure 1** Northern blot analysis of total RNA (10 µg in each well) for analysis of GSTπ transcripts. CaSki, MCF7/p, MCF7/DXR cell lines, normal cervical epitheliums (NC), invasive squamous cell carcinomas (T, primary tumour). No GSTπ transcript was detected in MCF7/p and a high level was found in MCF7/DXR as previously described (Batist *et al.*, 1986). Upper panel represents the transferred blots after the agarose gel was coloured with ethidium bromide (EB). Exposure time to Kodak XAR5 films was days.

**Figure 2** Slot blot analysis of total RNA from CaSki, MCF7/p, MCF7/DXR cell lines, normal cervical epithelium (NC), invasive squamous cell carcinomas of the uterine cervix (T, primary tumour; M, liver metastasis; LN, lymph node metastasis; R, recurrent tumour). Filter was hybridised using GSTπ probe. Three pairs of specimens obtained from the same patients were shown; NC-T (0.5 and 1.2 units respectively); T-M (3.5 and 8.4 units respectively); T-LN (2.9 and 2.5 units respectively). Filter was dehybridised and rehybridised using actin probe. Exposure time to Kodak XAR5 film was two days for both signals.

IV) (Table I). The GSTπ mRNA levels were not found to be significantly higher in recurrent tumours and lymph node metastases than in primary tumours. However, in the case of one liver metastasis treated with VCR, the GSTπ mRNA level was found higher (8.5 units) than that observed in the

**Figure 3** Levels of GSTπ mRNA in normal cervices (NC), carcinomas *in situ* (CIS), invasive squamous cell carcinomas (SCCa) of different clinical stages, 6 recurrent tumours (R) and 7 metastases (M) and invasive cervical cancers of other histological types (ADCa) (14 adenocarcinomas, one anaplastic cell carcinoma and one sarcoma). Specimens with GSTπ mRNA values > 1 unit (value found in CaSki cell line) were considered to contain overexpressed GSTπ gene.

untreated primary tumour (3.5 units) (Figure 2).

A previous immunohistochemical study (Shiratori *et al.*, 1987) had suggested that the human papillomavirus (HPV), a virus most likely involved in the carcinogenesis of cervical cancers (Zur Hausen, 1989), could favour the production of GSTπ in precancerous cervical lesions. Therefore we have analysed the GSTπ mRNA level in relation to the presence of HPV DNA sequences in tumours and normal cervical epitheliums. Of the 96 invasive cervical cancers for which both HPV detection (Riou *et al.*, 1990*a*) and GSTπ analysis could be done, no difference in GSTπ mRNA levels was found between HPV-positive (85% of tumours) and HPV-negative tumours. For epidermoid carcinomas, the mean levels of GSTπ mRNA were 2.6 U for both HPV-positive and HPV-negative tumours. For adenocarcinomas these mean levels were 1.6 and 2.2 for HPV-positive and HPV-negative tumours respectively. Moreover the GSTπ mRNA level was not found to be significantly higher in the four HPV-positive than in the 11 HPV-negative normal cervices. The normal cervical epithelium which displays more than 2 units (Figure 3) was HPV-negative.

*Analysis of tumoral DNA for GSTπ gene amplification*

Forty-nine preparations of genomic DNA from cervical cancers (40 samples) and normal cervices (nine samples) were analysed by Southern blot hybridisation using GSTπ probe. As expected, two DNA bands of 6.2 and 5.0 kb were observed (data not shown). No evidence for amplification of the GSTπ gene was found in the 49 DNA preparations analysed including those from tumours where the gene was overexpressed.

### Discussion

Cervical cancers are treated by surgery, radiation and cytotoxic drugs (Friedlander *et al.*, 1983; Haie *et al.*, 1988; McGuire *et al.*, 1989). Treatment depends on prognosis which is determined by clinicopathological parameters of which the most important are clinical stage at diagnosis and nodal status (Pejovic *et al.*, 1981). However in most cases, cervical cancers respond poorly to chemotherapy. Therefore, the present study was designed to test whether the GSTπ gene, suspected of involvement in drug resistance, was overexpressed in cervical cancer cells.

Using Northern blot hybridisation techniques we detected in the cervical tissues the expected 0.7 kb GSTπ transcript band (Figure 1). Transcripts were easily detectable in all the specimens analysed. When normalised to actin mRNA, GSTπ transcript levels in squamous cell carcinomas were found to undergo variations (range 0.5 to 8 units), but no significant difference was observed between cancers of different clinical stages suggesting that GSTπ is not associated with the progression of cervical cancers. In one liver metastasis the GSTπ mRNA level was found to be higher than in the primary tumour. This could however be due to the presence of normal tissue in the primary tumour. A significant difference of the frequency of tumours with GSTπ overexpression ($P < 0.01$) was found between squamous cell carcinomas and cancers of other histological types (adenocarcinomas, anaplastic cell carcinoma and sarcoma).

Our data confirmed previous immunohistochemical studies (Shiratori *et al.*, 1987) showing a GSTπ expression in about 90% of invasive cervical cancers. However they differ from those of Shiratori *et al.* (1987) since the presence of GSTπ transcripts was detected in all invasive cervical cancer as well as in all normal cervical epitheliums. Moreover we show that the presence of HPV DNA sequences in invasive cervical cancers and in normal cervical epitheliums does not influence the GSTπ mRNA level.

The overexpression of the GSTπ gene found in most invasive cervical cancers indicates that this gene is associated with the process of carcinogenesis. Such gene activation is more prevalent in squamous cell carcinomas than in adenocarcinomas. This is in accordance with the fact that squamous cell carcinomas are usually less sensitive to cytotoxic drugs than cancers of other histological types. In previous studies we have shown the presence of MDR1 transcripts at low, but significant levels in 43% of invasive cervical cancers and in 68% of normal cervical epitheliums (Riou *et al.*, 1990*b*) suggesting that expression of this gene may also be involved in the drug resistance phenotype of certain cervical cancers.

In conclusion, it is most likely that several mechanisms are involved in the drug resistance of invasive cervical cancers. The presence of high GSTπ mRNA levels may be a consequence of multiple biochemical alterations which accompany carcinogenesis and indirectly lead to a drug resistant phenotype.

We would like to thank Dr K. Cowan (Medicine Branch, NCI, Bethesda, USA) for his generous gifts of GSTπ cDNA probe and of MCF7/DXR cell line and Dr G. Orth (Laboratoire des Papillomavirus, Institut Pasteur, Paris) for data on HPV.

Supported by Association pour la Recherche sur le Cancer (ARC, Villejuif, France), Institut Gustave Roussy (Contrat de Recherche Clinique 90D10) and Fédération Nationale des Groupements des Entreprises Françaises dans la Lutte contre le Cancer.

### References

BATIST, G., TULPULE, A., SINHA, B.K., KATKI, A.G., MYERS, C.E. & COWAN, K.H. (1986). Overexpression of a novel anionic glutathione transferase in multidrug-resistant human breast cancer cells. *J. Biol. Chem.*, **261**, 15544.

BECK, W.T. (1989). Unknotting the complexities of multidrug resistance: the involvement of DNA topoisomerases in drug action and resistance. *J. Natl Cancer Inst.*, **81**, 1683.

D'ARPA, P. & LIU, L.F. (1989). Topoisomerase-targeting antitumor drugs. *Biochim. Biophys. Acta*, **989**, 163.

ENDICOTT, J.A. & LING, V. (1989). The biochemistry of P-glycoprotein mediated multidrug resistance. *Ann. Rev. Biochem.*, **58**, 137.

FRIEDLANDER, M., KAYE, S.B., SULLIVAN, A. & 10 others (1983). Cervical carcinoma: a drug-responsive tumor-experience with combined cisplatin, vinblastin and bleomycin therapy. *Gynecol. Oncol.*, **16**, 275.

GIOVANELLA, B.C., STEHLIN, J.S., WALL, M.F. & 5 others (1989). DNA topoisomerase I-targeted chemotherapy of human colon cancer in xenografts. *Science*, **246**, 1046.

HAIE, C., PEJOVIC, M.H., GERBAULET, A. & 12 others (1988). Is prophylactic para-aortic irradiation worthwhile in the treatment of advanced cervical carcinoma? Results of a controlled clinical trial of the EORTC radiotherapy group. *Radiother. Oncol.*, **11**, 101.

MANIATIS, T., FRITSCH, E.F. & SAMBROOK, J. (1982). *Molecular Cloning, A Laboratory Manual*, (eds). Cold Spring Harbor Laboratory, New York.

MCGUIRE, W.P., ARSENEAU, J., BLESSING, J.A. & 5 others (1989). A randomized comparative trial of carboplatin and iproplatin in advanced squamous carcinoma of the uterine cervix: a gynecologic oncology group study. *J. Clin. Oncol.*, **7**, 1462.

MOSCOW, J.A. & COWAN, K.H. (1988). Multidrug resistance. *J. Natl Cancer Inst.*, **80**, 14.

MOSCOW, J.A., TOWNSEND, A.J., GOLDSMITH, M.E. & 6 others (1988). Isolation of the human anionic glutathione S-transferase cDNA and the relation of its gene expression to estrogen-receptor content in primary breast cancer. *Proc. Natl Acad. Sci. USA*, **85**, 6518.

PASTAN, I. & GOTTESMAN, M.M. (1987). Multiple drug resistance in human cancer. *New Engl. J. Med.*, **316**, 1388.

PEJOVIC, M.H., WOLFF, J.P., KRAMAR, A. & GOLDFARB, E. (1981). Cure rate estimation and long-term prognosis of uterine cervix carcinoma. *Cancer*, **47**, 203.

RIOU, G., FAVRE, M., JEANNEL, D., BOURHIS, J., LE DOUSSAL, V. & ORTH, G. (1990*a*). Association between poor prognosis in early-stage invasive cervical carcinomas and non-detection of HPV DNA. *Lancet*, **335**, 1171.

RIOU, G.F., ZHOU, D., AHOMADEGBE, J.C., GABILLOT, M., DUVILLARD, P. & LHOMME, C. (1990*b*). Expression of multidrug-resistance (MDR1) gene in normal epithelia and in invasive carcinomas of the uterine cervix. *J Natl Cancer Inst.*, **82**, 1493.

SHENG, Z.M., BARROIS, M., KLIJANIENKO, J., MICHEAU, C., RICHARD, J.M. & RIOU, G. (1990). Analysis of the c-Ha-*ras*-1 gene for deletion, mutation, amplification and expression in lymph node metastases of human head and neck carcinomas. *Br. J. Cancer*, **62**, 398.

SHIRATORI, Y., SOMA, Y., MARUYAMA, H., SATO, S., TAKANO, A. & SATO, K. (1987). Immunohistochemical detection of the placental form of glutathione S-transferase in dysplastic and neoplastic human uterine cervix lesions. *Cancer Res.*, **47**, 6806.

TEW, K.D. (1989). The involvement of glutathione S-transferases in drug resistance. In *Anticancer drugs*, Tapiero, H., Robert, J. & Lampidis, T.J. (eds) vol. 191, p. 103. Colloque INSERM/John Libbey.

ZUR HAUSEN, H. (1989). Papillomaviruses in anogenital cancer as a model to understand the role of viruses in human cancers. *Cancer Res.*, **49**, 4677.